# ^68^Ga-DOTATATE Prepared from Cyclotron-Produced ^68^Ga: An Integrated Solution from Cyclotron Vault to Safety Assessment and Diagnostic Efficacy in Neuroendocrine Cancer Patients

**DOI:** 10.2967/jnumed.121.263768

**Published:** 2023-02

**Authors:** Sébastien Tremblay, Jean-François Beaudoin, Ophélie Bélissant Benesty, Samia Ait-Mohand, Véronique Dumulon-Perreault, Étienne Rousseau, Éric E. Turcotte, Brigitte Guérin

**Affiliations:** 1 Department of Nuclear Medicine and Radiobiology, Université de Sherbrooke, Sherbrooke, Quebec, Canada; and; 2 Sherbrooke Molecular Imaging Center of the CRCHUS, Sherbrooke, Quebec, Canada

**Keywords:** cyclotron ^68^Ga, in-vault dissolution system, ^68^Ga-DOTATATE, PET imaging, cancer patients

## Abstract

Cyclotron production of ^68^Ga is a promising approach to supply ^68^Ga radiopharmaceuticals. To validate this capability, an integrated solution for a robust synthesis of ^68^Ga-DOTATATE prepared from cyclotron-produced ^68^Ga was achieved. A retrospective comparison analysis was performed on patients who underwent PET/CT imaging after injection of DOTATATE labeled with ^68^Ga produced by a cyclotron or eluted from a generator to demonstrate the clinical safety and diagnostic efficacy of the radiopharmaceutical as a routine standard-of-care diagnostic tool in the clinic. **Methods:** An enriched pressed ^68^Zn target was irradiated by a cyclotron with a proton beam set at 12.7 MeV for 100 min. The fully automated process uses an in-vault dissolution system in which a liquid distribution system transfers the dissolved target to a dedicated hot cell for the purification of ^68^GaCl_3_ and radiolabeling of DOTATATE using a cassette-based automated module. Quality control tests were performed on the resulting tracer solution. The internal radiation dose for ^68^Ga-DOTATATE was based on extrapolation from rat biodistribution experiments. A retrospective comparison analysis was performed on patients who underwent PET/CT imaging after injection of DOTATATE labeled with cyclotron- or generator-produced ^68^Ga. **Results:** The synthesis of ^68^Ga-DOTATATE (20.7 ± 1.3 GBq) with high apparent molar activity (518 ± 32 GBq/μmol at the end of synthesis) was completed in 65 min, and the radiopharmaceutical met the requirements specified in the *European Pharmacopoeia* monograph on ^68^Ga-chloride (accelerator-produced) solution for radiolabeling. ^68^Ga-DOTATATE was stable for at least 5 h after formulation. The dosimetry calculated with OLINDA for cyclotron- and generator-produced ^68^Ga-DOTATATE was roughly equivalent. The SUV_mean_ or SUV_max_ of tumoral lesions with cyclotron-produced ^68^Ga-DOTATATE was equivalent to that with generator-produced ^68^Ga. Among physiologic uptake levels, a significant difference was found in kidneys, spleen, and stomach wall, with lower values in cyclotron-produced ^68^Ga-DOTATATE in all cases. **Conclusion:** Integrated cyclotron production achieves reliable high yields of clinical-grade ^68^Ga-DOTATATE. The clinical safety and imaging efficacy of cyclotron-produced ^68^Ga-DOTATATE in humans provide supporting evidence for its use in routine clinical practice.

## Visual Abstract

**Figure:**
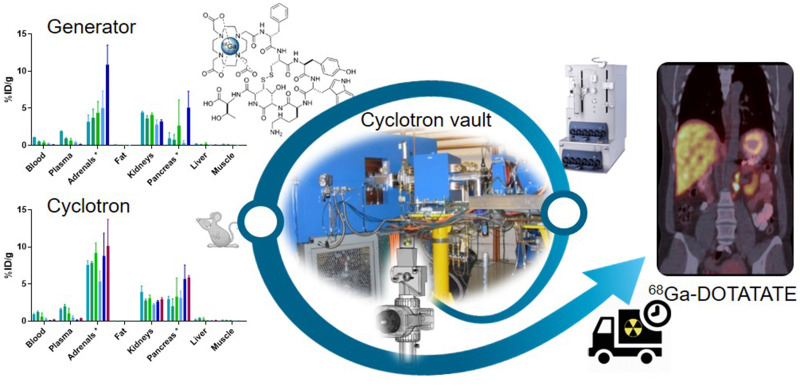

Commercial production of the ^68^Ge/^68^Ga generator increased accessibility and kick-started metal radiolabeling of peptides for medical diagnosis. The demand for ^68^Ga now greatly exceeds the production capacity of generators (1), and the use of cyclotrons for production of ^68^Ga by a ^68^Zn(p,n)^68^Ga reaction at energies of 12–14 MeV on a larger scale is becoming a necessity.

Use of cyclotrons for the production of ^68^Ga first expanded with liquid targets for a yield increase of 10 times generator production, with the convenience of enriched ^68^Zn recycling and compatibility with existing distribution systems for the liquid targets (2–4). However, problems of target density, high pressure, and metal contamination by the targets limit the maximum production quantity and labeling efficiency (2–4). The use of solid targets allows much higher yields from 50 to 100 times generator capacity. However, its spread to different sites is limited by the complexity of target production, the recovery of the solid target while avoiding a high dose for handling, and the complex, expensive systems required for transfers from the cyclotron vault to the units of synthesis (5–11).

The aim of this study was to give a complete high-yield integrated solution, from simple target preparation, irradiation, and dissolution to production of good-manufacturing-practice–compliant ^68^Ga-DOTATATE. To take advantage of the higher production capacity of cyclotrons with solid targets, we first investigated the quality of ^68^Ga produced at 12.7 MeV to demonstrate robustness in the production of multiple doses of ^68^Ga-DOTATATE. The chemical and radiochemical purity and the dosimetry of this radiopharmaceutical were examined for human use. A phase 3 study aiming to evaluate the innocuity and safety profile of ^68^Ga-DOTATATE prepared from ^68^Ga produced by a cyclotron was initiated to establish the procedure as a routine standard-of-care diagnostic tool for all neuroendocrine cancer patients. This was a single-center study but with recruitment across all of Canada. The trial was prospective, nonrandomized, and open-label and had no control group (ClinicalTrials.gov identifier NCT04847505, approved by Health Canada). From this study, a retrospective comparison analysis was performed on patients who underwent PET/CT imaging after injection of DOTATATE labeled with ^68^Ga produced by a cyclotron or eluted from a generator (ClinicalTrials.gov identifier NCT02810600, a completed phase 2 study with 2,120 participants).

## MATERIALS AND METHODS

^68^Zn metal powder (≥98.1% enriched) was purchased from ISOFLEX USA and Neonest AB. Nitric acid, 70% (≥99.999% trace metals basis); hydrochloric acid, 37% (≥99.999% trace metals basis); ammonium formate (≥99.995% trace metals basis); sodium phosphate dibasic (≥99.99% trace metals basis); potassium phosphate monobasic (99.99% trace metals basis); acetonitrile; hydroxylamine hydrochloride, 99% (ReagentPlus); 2,3,5,6-tertrafluorophenol; and sodium hydroxide, 98% (of American Chemical Society grade) were purchased from Aldrich Chemical. Ascorbic acid (≥99.99998% trace select) was obtained from Honeywell. All solutions and dilutions were prepared with Optima (Sorbent Technologies) ultra-performance liquid chromatography/high-performance liquid chromatography water. Methanol of high-performance liquid chromatography grade and NaCl were purchased from Fisher Scientific. The reverse-phase cartridge and Accell Plus CM cationic exchange resin were purchased from Waters. Empty cartridges were purchased from UCT, and *N*-(3-dimethylaminopropyl)-*N*′-ethylcarbodiimide hydrochloride was from Matrix Innovation. DOTATATE was obtained from AUSPEP. The radio thin-layer chromatography (TLC) was performed using instant-TLC silica gel paper from Agilent and a radio-TLC scanner (Bioscan AR-2000). γ-ray spectrometry was conducted on a high-purity germanium detector (GMX; Ortec) calibrated with a National Institute of Standards and Technology–traceable γ-set (^133^Ba, ^109^Cd, ^57^Co, ^60^Co, ^137^Cs, ^54^Mn, and ^22^Na) from Eckert and Ziegler Isotope Products. The pH strips (range, 2–10) were purchased from Millipore-Sigma, and Quantofix (Macherey-Nagel) iron and zinc test strips were from Aldrich. Hydroxamate resin was prepared from the modified Accell Plus CM cationic exchange resin using the procedure developed by Verel et al. (12) and was packed in a 1-mL cartridge (United Chemical Technologies). Benzene sulfonic resin (CUBCX123 and CUBCX111) was bought from UCT. A IGGl00 ^68^Ge/^68^Ga generator was obtained from Eckert and Ziegler EUROTOPE.

### Target Preparation

The target preparation was already described by Alnahwi et al. (6). Briefly, isotopically enriched ^68^Zn powder was pressed in a die of 8 mm (155 mg) by a hydraulic press (module 3912; Carver) and deposed in the appropriate cavity diameter of the magnetic target carrier (6).

### Irradiation Procedure

Targets were irradiated facing a perpendicular proton beam in a solid target holder, TA-1186D (ACSI), mounted to a target selector installed directly on a TR-19 or TR-24 cyclotron (Advanced Cyclotron Systems) in conjunction with our customized magnetic target carrier (6). Beam degradation was achieved by the combined density of a tantalum foil (125–150 μm; Goodfellow) and the target carrier. The resulting energy was determined using the SRIM 2013 simulator (13). For irradiation, the incident energy beam was set to 18.2 MeV and degraded to 12.7 MeV with the aluminum degrader of 0.4 mm to minimize formation of ^67^Ga by the nuclear reaction ^68^Zn(p,2n)^67^Ga (14), with a target current of 20 μA applied during approximately 100-min irradiations on both cyclotrons.

### In-Vault Dissolution System

**FIGURE 1.**
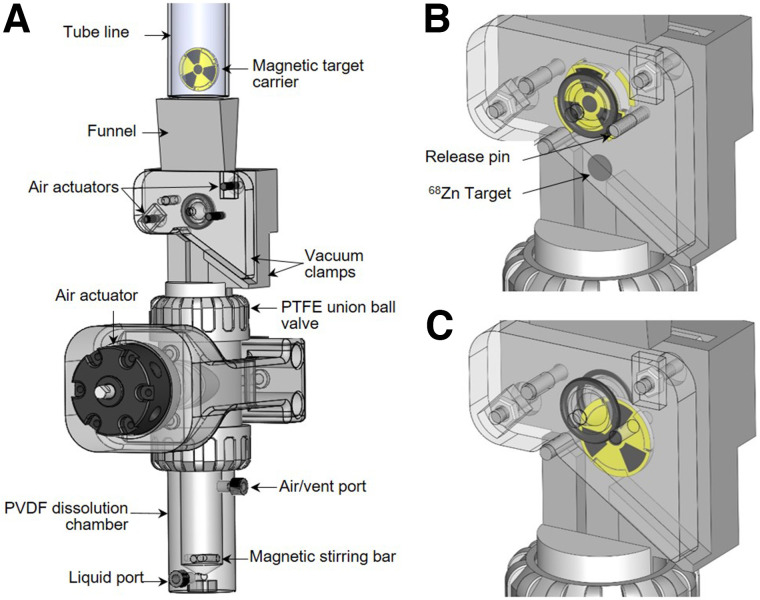
In-vault dissolution system: component assembly (A), ^68^Zn-enriched target release (B), and magnetic target carrier release (C). PTFE = polytetrafluoroethylene; PVDF = polyvinylidene fluoride.

After the irradiation in the cyclotron target holder, the target carrier was released down a tube line into a dissolution system (Fig. 1). This custom-built system is located in the cyclotron vault and remotely automated by an industrial programmable logic controller. The top portion of the system funnels the target carrier into an air-activated vacuum clamp that opens and closes the magnetic target carrier to release the ^68^Zn target payload into the polyvinylidene fluoride dissolution chamber. The middle section gates the passage of the target to the dissolution chamber with an air-activated union ball valve (19-mm opening) that seals off the chamber during the target dissolution process. The final portion of the system is the dissolution chamber, where an air/vent port (Fig. 1A) and an air-activated distribution valve (VICI) govern the incoming injections of 1.5 mL of 7 M nitric acid, 3 mL of water, and 2.75 mL of 2.5 M ammonium formate buffering solution through the liquid port (Fig. 1A) during the dissolution sequence and selects the destination line of the dissolved target to the proper synthesis unit. Inside the chamber, a magnetic stirring bar is activated for 2 min for complete dissolution (6), after which water and ammonium formate were added. The dissolved target solution arrives about 7 min after the cyclotron irradiation in the destination hot cell. With the process completed, the vacuum clamp and air-actuated release pin (Fig. 1B) allow the magnetic target carrier to exit the system via the ejection slide (Fig. 1C). At this point, all valves are reset, and the system is ready for another combined synthesis–dissolution process to target water flushing; the overall step is accomplished in 20 min after the end of bombardment.

### Peptide Radiolabeling

**FIGURE 2.**
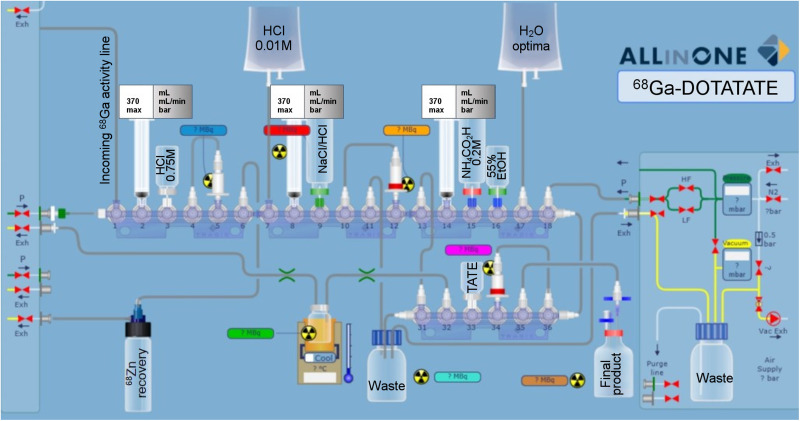
AllinOne schematic of ^68^Ga-DOTATATE synthesis, including ^68^GaCl_3_ purification. Exh = exhaust; mbar = millibar; vac = vacuum.

The ^68^GaCl_3_ purification (6) and peptide radiolabeling steps were both performed on an AllInOne automated module (TRASIS), as shown in Figure 2. The ^68^GaCl_3_ purification was performed using the optimized procedure described by Alnahwi et al. (6). After the transfer of ^68^Ga, the line was rinsed with 0.01 M HCl (0.5 mL) for maximum recovery. The pH was adjusted to 3.5 with 1.2 mL of a 0.2 M ammonium formate metal trace buffer solution. The buffered solution was transferred to the reactor prefilled with a 1-mL solution of 60 μg of DOTATATE and 25 mg of ascorbic acid of traceSELECT (Sigma-Aldrich) grade. After mixing with nitrogen, the pH was 3.4–3.8 and the reaction mixture was raised to 100°C during 13 min. The radiolabeling yield was greater than 98%. After the labeling step, the reactor was cooled to 50°C, and the solution was then drawn into a syringe prefilled with 5 mL of water at room temperature. The peptide solution was passed through 500-mg C18 reversed-phase resin, and the reactor was rinsed with an extra 4 mL of water. The column was then washed twice with 10 mL of water. The ^68^Ga-DOTATATE was recovered with 3.5 mL of 55% (v/v) ethanol/water solution through the C18 column to the product vial for final formulation. The formulation was achieved by adding 17.5 mL of solution containing 0.14 g of Na_2_HPO_4_, 0.024 g of KH_2_PO_4_, 0.1 g of NaCl, and 100 mg of ascorbic acid to the product vial for a total volume of 21 mL and 9.4% ethanol. This solution was filtered through a sterile 0.22-μm polyvinylidene fluoride membrane filter (Millipore) and fractionized with the Eckert and Ziegler module in the clean room.

### Quality Control Tests

Quality control tests were performed on formulated ^68^Ga-DOTATATE. The pH was measured with pH strips. The radiochemical identity and purity were determined by ultra-high-performance liquid chromatography (Acquity; Waters) with an evaporative light-scattering detector, a flow-count radiodetector (Bioscan), and instant TLC. Samples (1 μL) were injected and analyzed on an Acquity BEH C18 column (1.7 μm, 2.1 × 50 mm) and compared with a home-made nonradioactive gallium-DOTATATE standard. The instant-TLC silica gel paper was eluted with a 77 g/L solution of ammonium acetate:MeOH (1:1). The radionuclidic purity was verified by γ-ray spectrometry on a calibrated high-purity germanium detector with a zoom energy window of 1–2,000 keV. Samples were counted for 2 min after the end of synthesis (EOS). In addition, the tests were repeated at 16–24 h after EOS to quantify radionuclidic impurities (^67^Ga [half-life, 3.26 d] and ^66^Ga [half-life, 9.49 h]). Chemical purity was evaluated using commercially available indicator strips to measure iron and zinc in the formulated ^68^Ga-DOTATATE. The endotoxin levels were assayed by the *Limulus* amebocyte lysate method with an Endosafe-PTS test system (Charles River Laboratories International). Sterility tests were performed by a licensed laboratory (Nucro-Technics).

### Animals

All animal studies were conducted in compliance with the Canadian Council on Animal Care guidelines and with the approval of the Animal Care Committee of the Université de Sherbrooke.

### Biodistribution Studies

Biodistribution studies were conducted on 12-wk-old Fisher female rats weighing 150–175 g (Charles River) to determine the uptake of ^68^Ga-DOTATATE in various organs. A 7- to 14-MBq dose of cyclotron- or generator-produced ^68^Ga-DOTATATE was injected into the tail vein of the isoflurane-anesthetized rats. At 15, 30, 45, 60, or 120 min after injection, while anesthesia was maintained, blood was taken by cutting the femoral artery. The animals were then killed by CO_2_ inhalation and the organs of interest were removed, rinsed, and blotted dry before the radioactivity was counted in a Hidex γ-counter. The results were expressed as percentage injected dose per gram of tissue.

### Clinical PET Imaging

A retrospective comparison analysis was performed on patients who underwent PET/CT imaging after injection of DOTATATE labeled with cyclotron- or generator-produced ^68^Ga. The institutional ethic board approved the study, and all subjects gave written informed consent.

Patients with progressive disease between the 2 examinations (defined by at least one new lesion) or examinations performed with different cameras were excluded.

PET/CT imaging was performed from head to mid thigh, on a Gemini TF or Gemini GXL PET/CT scanner (Philips). An unenhanced CT scan was obtained using the following parameters: slice thickness, 3 mm; increment, 3 mm; 120 kVp, and 55–83 mAs, depending on the patient’s weight. Immediately after CT scanning, whole-body PET was performed in 3-dimensional mode (matrix, 144 × 144). For each bed position (15 cm; overlapping scale, 5 cm), a 2-min acquisition with a 57.6-cm field of view was used.

The emission data were corrected for decay and for random and scatter events. Reconstruction used the 3-dimensional row-action maximum-likelihood algorithm with 2 iterations, a relaxation parameter of 0.5, and a 2-mm-radius spherically symmetric basis function (blobs). Attenuation was corrected using the low-dose unenhanced CT data. Image analysis was performed using OASIS software (Segami).

To compare both cyclotron- and generator-produced ^68^Ga-DOTATATE, regions of interest were drawn on transaxial slices around areas of focal uptake in the pituitary gland; lacrimal, parotid, submandibular, and sublingual salivary glands; nasal mucosa; thyroid gland; mediastinal blood pool (aortic arch); adrenals; liver; spleen; stomach wall; bowel; kidney cortex; uncinate process; and gluteal musculature (as background). Isocontour volumes at 70% of the maximum pixel value were drawn automatically, and the SUV_mean_ and SUV_max_ were measured in all these volumes. The SUV_mean_ and SUV_max_ of neuroendocrine lesions were also recorded for up to 10 lesions per patient.

Cyclotron- and generator-produced ^68^Ga-DOTATATE values were compared using Wilcoxon matched-pairs rank tests for each patient. A *P* value of less than 0.05 was considered statistically significant.

## RESULTS

### Target Preparation and Irradiation

The ^68^Zn-pressed target irradiation challenge was to manage the low melting point of the zinc (419.5°C) and the focused beam on the solid target. To avoid target overheating at low current, a 125-μm tantalum foil was efficiently used to diffuse the beam and reduce the proton energy to 12.7 MeV on the target.

### ^68^Ga-DOTATATE Preparation

A fully automated dissolution system was developed to facilitate the radiosynthesis of ^68^Ga for large-scale and routine production using a pressed ^68^Zn target. The activity of the transferred ^68^GaCl_3_ solution from the vault to the hot cell was 46.2 ± 2.2 GBq (56.6 GBq at the end of bombardment), with a saturated yield of 4.4 ± 0.1 GBq/μA when using the 8-mm-diameter ^68^Zn target irradiated at 12.7 MeV for 100.3 ± 2.4 min at 20 ± 0.4 μA. The ^68^GaCl_3_ was purified using the optimized procedure described by Alnahwi et al. (6).

DOTATATE aliquots (60 μg) formulated in water (300 μL) were stable for up to 30 d when the solution was kept frozen at −20°C. To avoid radiolysis at high activity levels, the peptide precursor was loaded in the reaction vessel in the presence of ascorbic acid, and the pH was adjusted before transfer of the purified ^68^GaCl_3_ solution. A mean of 20.7 ± 1.3 GBq (*n* = 14) of ^68^Ga-DOTATATE at EOS was produced in less than 35 min, for a global decay-corrected yield of 66% ± 5% (*n* = 14). The final formulation, filtration, and distribution process was performed within 10–15 min.

The estimated apparent molar activity at EOS was 518 ± 32 GBq/μmol (*n* = 14), which is 20-fold higher than the 25 GBq/μmol reported by Thisgaard et al. (11) for cyclotron-produced ^68^Ga-DOTATATE. From our previous studies, new generators produce 0.665 ± 0.043 GBq (*n* = 10) of ^68^Ga-DOTATATE that has an apparent molar activity of 63 ± 13 GBq/μmol with a performance that decays over time.

### Quality Control Results

Samples of all productions (*n* = 14, Table 1) complied with all specifications. ^67^Ga and ^66^Ga contents were, respectively, 0.052% ± 0.004% and 0.017% ± 0.006% at EOS (*n* = 14).

**TABLE 1.** Quality Control Results for ^68^Ga-DOTATATE

Analysis	Method	Specifications EOS	Results (*n* = 14)
Appearance	Visual	Clear, no color	Pass
pH	pH strip	4.0–8.0*	5.4 ± 0.2
Peptide DOTATATE	Calculation	≤60 μg*	57.3 ± 0.5 μg
Radiochemical identity	Ultra-performance liquid chromatography retention time	±10%	0.90% ± 0.05%
Radiochemical purity	(100-A) × T = %	≥91%	98.4% ± 0.9%
Radionuclidic purity	γ-511 and 1,077 keV	≥98%	99.7% ± 0.3%
Radionuclide identification	Half-life	62–74 min*	67.5 ± 0.5 min
Pyrogenicity	Inoculation	≤8.3 IU/mL*	Pass
Filter integrity	Bubble point	≥345 kPa (50 psi)	Pass
Zinc/iron	Strips	≤10 μg/GBq	<1 μg/GBq
^67^Ga and ^66^Ga contents	γ-ray analysis	≤2%	^67^Ga ≤ 0.052% ± 0.004%^†^ ^66^Ga ≤ 0.017% ± 0.006%^†^

* Our release criteria.

^†^ Values recalculated to moment of EOS from results obtained 16–24 h after EOS.

A = percentage of radioactivity due to ^68^Ga impurity (retardation factor = 0–0.1) determined by instant TLC; T = proportion of the radioactivity in the area of the peak due to ^68^Ga-DOTATATE relative to the total areas of the peaks in the chromatogram obtained from ultra-high-performance liquid chromatography analysis.

### Assessment of Internal Radiation Dose

**FIGURE 3.**
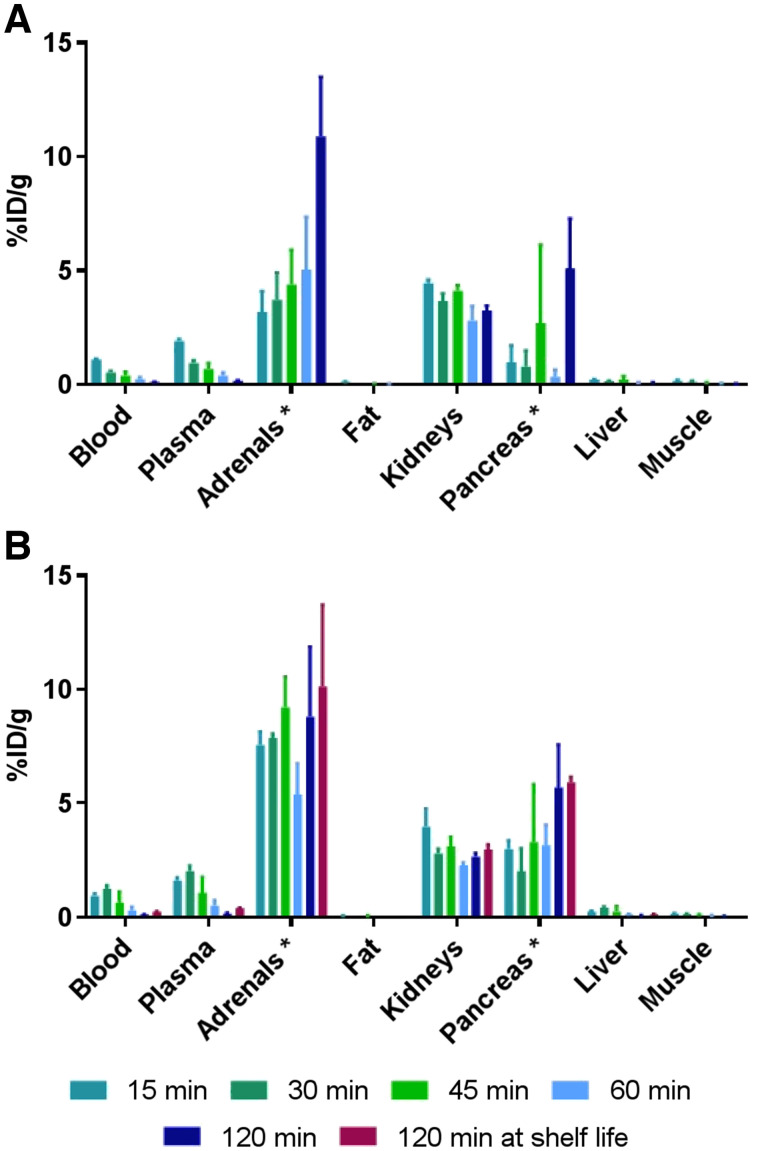
Biodistribution in rats of DOTATATE labeled with generator-produced (A) and cyclotron-produced (B) ^68^Ga at various time points. %ID = percentage injected dose.

To estimate the dosimetry of the ^68^Ga-DOTATATE, biodistribution experiments were conducted on female Fischer rats at different time points after injection (Fig. 3). Cyclotron-produced ^68^Ga-DOTATATE showed significantly higher uptake in the adrenals and pancreas, organs of interest rich in somatostatin receptors, at early time points (15 and 30 min, *P* < 0.005) after injection. A potential explanation is that the cyclotron-produced ^68^Ga-DOTATATE has the larger apparent molar activity. Cyclotron-produced ^68^Ga-DOTATATE and generator-produced ^68^Ga-DOTATATE were biologically equivalent at late time points, giving identical kinetic and biodistribution patterns in animals.

Dosimetry extrapolated to humans was computed using OLINDA/EXM (Vanderbilt University, 2003) from the residence times scaled to humans. Since female rats were used for the biodistribution experiments, the adult female model provided by the software was applied for computations. Extrapolated dosimetry is detailed in Table 2 as absorbed dose.

**TABLE 2.** Dosimetry Extrapolated to Humans for ^68^Ga-DOTATATE

	Absorbed dose (mGy/MBq)	
Tissue	Generator ^68^Ga	Cyclotron ^68^Ga
Adrenals	1.33e−01	1.52e−01
Brain	5.45e−04	7.41e−04
Breasts	8.99e−04	9.52e−04
Gallbladder wall	1.66e−03	1.85e−03
Lower large intestine wall	9.41e−04	9.78e−04
Small intestine	1.21e−03	1.20e−03
Stomach wall	1.42e−03	1.79e−03
Upper large intestine wall	1.14e−03	1.14e−03
Heart wall	2.52e−02	2.69e−02
Kidneys	9.01e−02	6.29e−02
Liver	4.42e−03	5.30e−03
Lungs	1.12e−02	1.17e−02
Muscle	3.23e−03	3.41e−03
Ovaries	7.95e−03	4.94e−03
Pancreas	1.36e−02	7.28e−02
Red marrow	6.46e−03	6.68e−03
Osteogenic cells	7.25e−03	8.96e−03
Skin	6.10e−04	6.35e−04
Spleen	5.42e−03	5.79e−03
Thymus	1.61e−03	1.70e−03
Thyroid	7.12e−04	7.60e−04
Bladder wall	7.51e−04	7.83e−04
Uterus	5.28e−03	6.67e−03
Total body	2.83e−03	3.00e−03

Estimated ^68^Ga-DOTATATE dosimetry is acceptable when compared with other PET tracers in use in humans and shows that dosimetry for ^68^Ga-DOTATATE synthesized from cyclotron-produced ^68^Ga and generator-produced ^68^Ga is roughly equivalent. The main difference in calculated dosimetry between the 2 tracers is in the pancreas, kidneys, and adrenals.

### Clinical Studies

PET/CT examinations after injection of cyclotron- and generator-produced ^68^Ga-DOTATATE were compared for 12 patients (14 lesions): 4 using the Gemini GXL scanner and 8 the Gemini TF PET/CT scanner. The mean interval between the examinations was 373.5 ± 315.8 d.

Cyclotron production allowed injection of a higher radiotracer activity than did generator production (3.9 ± 1.0 MBq/kg and 1.9 ± 0.6 MBq/kg, respectively; *P* = 0.01). There was no difference in injection-to-acquisition interval between the examinations (69.5 ± 11.9 and 65.4 ± 11.6 min, respectively; *P* = 0.34).

**FIGURE 4.**
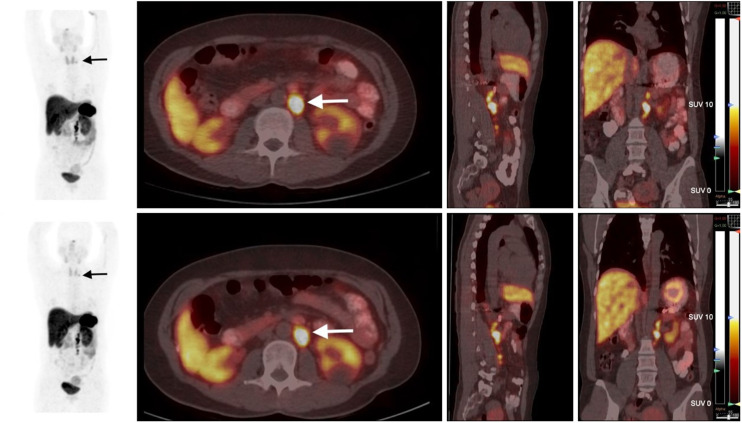
Example of 2 examinations, with cyclotron-produced (top row) and generator-produced (bottom row) ^68^Ga-DOTATATE PET/CT. From left to right in each row maximum-intensity projection (MIP), axial, sagittal and coronal planes. Examinations were performed 16 mo apart on same patient, who was being followed after removal of left pheochromocytoma but had secondary supraclavicular (black arrows) and retroperitoneal (white arrows) node metastasis.

SUV_max_ and SUV_mean_ are compared in Tables 3 and 4, respectively. The SUV_mean_ and SUV_max_ of tumoral lesions did not differ between cyclotron and generator production. Regarding physiologic uptake, a significant difference was found in the kidneys, spleen, and stomach wall, with lower values in cyclotron-produced ^68^Ga-DOTATATE in all cases (Fig. 4). All these organs are large and highly somatostatin receptor–expressing (15), but to our knowledge, the effect of injected activity or apparent molar activity on physiologic uptake in somatostatin receptor PET imaging has not been described.

**TABLE 3.** Comparison of SUV_max_ of Physiologic and Tumoral Uptake Between Cyclotron- and Generator-Produced ^68^Ga-DOTATATE

	SUV_max_		
Tissue	Cyclotron ^68^Ga	Generator ^68^Ga	*P*
Pituitary	4.56 ± 1.57	5.13 ± 1.54	0.3291
Liver	8.33 ± 2.19	8.95 ± 2.47	0.3804
Thyroid	4.25 ± 2.03	4.25 ± 2.11	>0.9999
Parotid	2.37 ± 1.01	2.41 ± 1.04	0.8657
Submandibular	2.77 ± 1.21	2.87 ± 1.17	0.6079
Nasal mucosa	2.16 ± 0.54	2.34 ± 0.45	0.9463
Aortic arch	0.95 ± 0.36	0.97 ± 0.20	0.6221
Kidneys	14.30 ± 5.23	16.76 ± 4.12	0.0024*
Spleen	22.79 ± 5.29	26.44 ± 5.33	0.0015*
Uncinate process	5.31 ± 2.45	5.96 ± 2.88	0.1475
Stomach wall	6.30 ± 3.36	8.69 ± 2.37	0.0093*
Bowel	6.91 ± 1.94	7.96 ± 1.92	0.1294
Adrenal	11.09 ± 4.41	12.44 ± 4.39	0.0923
Gluteal musculature	1.19 ± 0.49	1.31 ± 0.33	0.292
Tumors	9.13 ± 6.18	7.94 ± 5.61	0.346

* *P* value ≤ 0.05.

Data are mean ± SD.

**TABLE 4.** Comparison of SUV_mean_ of Physiologic and Tumoral Uptake Between Cyclotron- and Generator-Produced ^68^Ga-DOTATATE

	SUV_mean_		
Tissue	Cyclotron ^68^Ga	Generator ^68^Ga	*P*
Pituitary	3.82 ± 1.34	4.32 ± 1.31	0.457
Liver	6.56 ± 1.67	6.95 ± 1.81	0.4131
Thyroid	3.43 ± 1.65	3.42 ± 1.72	>0.9999
Parotid	1.89 ± 0.83	1.91 ± 0.82	0.8657
Submandibular	2.22 ± 0.96	2.31 ± 0.95	0.6377
Nasal mucosa	1.75 ± 0.45	1.85 ± 0.33	0.8945
Aortic arch	0.76 ± 0.29	0.79 ± 0.19	0.5879
Kidneys	11.09 ± 4.07	13.00 ± 3.36	0.0049*
Spleen	18.72 ± 4.24	21.41 ± 4.14	0.002*
Uncinate process	4.25 ± 1.56	4.80 ± 2.29	0.083
Stomach wall	5.04 ± 2.74	6.96 ± 1.87	0.0093*
Bowel	5.49 ± 1.56	6.33 ± 1.48	0.1099
Adrenal	9.01 ± 3.59	10.05 ± 3.55	0.1133
Gluteal musculature	0.97 ± 0.39	1.09 ± 0.28	0.207
Tumors	5.14 ± 5.42	6.52 ± 4.59	0.583

* *P* value ≤ 0.05.

Data are mean ± SD.

## DISCUSSION

In recent years, several groups, including ours, have developed new liquid and solid targets and robust separation procedures to manufacture high-purity ^68^Ga with cyclotrons (2–11). As previously described by Alnawhi et al. (6), the preparation and assembly of ^68^Zn-pressed target was easy, time-effective for serial production, and inexpensive. There is clear and convincing evidence that the quality of cyclotron-produced ^68^Ga is comparable to that of generator-produced ^68^Ga (2–11) and that large quantities can be prepared (5*,*^6^*,*^10^*,*^11^), which enable transportation of ^68^Ga tracer. ^68^Ga-DOTATATE can safely be used up to 5 h after EOS, when the combined value for ^66^Ga and ^67^Ga content reaches 1.25% of the activity of ^68^Ga as compared with the 2% limit prescribed in the *European Pharmacopoeia* monograph (16).

The lower physiologic uptake and similar tumor uptake obtained with ^68^Ga-DOTATATE produced by cyclotron could result in only a better tumor-to-background ratio in these areas. For the last 18 mos, a weekly production of ^68^Ga-DOTATATE was successfully performed and more than 1,075 clinical scans were done. One should note that a single cyclotron production of ^68^Ga-DOTATATE allowed for scheduling of 10 patients on 2 simultaneously running PET scanners, compared with 2 patients for generator-produced ^68^Ga-DOTATATE.

The present clinical trial confirmed that ^68^Ga-DOTATATE prepared from cyclotron-produced ^68^Ga is safe and provides diagnostic efficacy equivalent to that of the radiopharmaceutical prepared from generator-produced ^68^Ga.

## CONCLUSION

We propose a complete high-yield integrated solution for cyclotron-produced and good-manufacturing-practice–compliant ^68^Ga-DOTATATE. Use of the in-vault dissolution system combines the high-yield production of the solid target with the versatility of the liquid-target distribution to multiple synthesis units. The results of the present study further support adoption of cyclotron-produced ^68^Ga-DOTATATE in clinical practice.

## DISCLOSURE

Étienne Rousseau, Éric Turcotte, and Brigitte Guérin are members of the Centre de recherche du CHUS, funded by the Fonds de recherche du Québec–Santé. Brigitte Guérin is the holder of the Jeanne and J.-Louis Lévesque Chair in Radiobiology at the Université de Sherbrooke. This work was partially financed by Oncopole, which receives funding from Merck Canada Inc. and from Fonds de recherche du Québec–Santé, as well as from the Cancer Research Society. One Patent Cooperation Treaty patent was filed for some of the material presented in this article. No other potential conflict of interest relevant to this article was reported.

## Notes

KEY POINTS **QUESTION:** How does the development of cyclotron-produced ^68^Ga-DOTATATE facilitate PET imaging of neuroendocrine cancer patients? **PERTINENT FINDINGS:** A robust and good-manufacturing-practice–compliant synthesis of ^68^Ga-DOTATATE from cyclotron-produced ^68^Ga was achieved, expanding the imaging time window and the number of patients per production compared with generator-based ^68^Ga-DOTATATE. We also showed that cyclotron-produced ^68^Ga-DOTATATE is safe for clinical use. **IMPLICATIONS FOR PATIENT CARE:** Cyclotron-produced ^68^Ga-DOTATATE can provide high-contrast detection of neuroendocrine tumors with reduced physiologic uptake in highly somatostatin receptor–expressing organs.
